# Bilateral thalamic glioma in a 6-year-old child

**DOI:** 10.4103/1817-1745.66672

**Published:** 2010

**Authors:** Dinesh K. Rajput, Anant Mehrotra, Arun K. Srivastav, Raj Kumar, Ashok K. Mahapatra

**Affiliations:** Department of Neurosurgery, Sanjay Gandhi Post Graduate Institute of Medical Sciences, Lucknow, India

**Keywords:** Bithalamic gliomas, thalamic gliomas

## Abstract

Bithalamic gliomas are extremely rare tumors of central nervous system. Although they are usually benign in nature, their outcome is poor because of the involvement of thalamic nuclei and inadequate surgical excision. Surgery is usually done to get tissue for diagnosis. Role of radiotherapy and chemotherapy is questionable. They are unique in their metabolic and neuroradiological properties. We report herein a 6-year-old male of bithalamic astrocytoma (WHO grade 2) who presented with raised intracranial pressure and tremors in right upper limb. The child had a very huge bithalamic mass which was debulked through the interhemispheric transcallosal approach in order to reduce the mass effect. He had a stormy post-operative course to recover gradually.

## Introduction

Bilateral thalamic glioma are rare tumors of brain. Though they are benign in nature but their outcome is poor in comparison to unilateral thalamic glioma. They grow slowly and present with huge size involving nuclei of thalamus. Surgery had a limited role like biopsy, management of hydrocephalus and decompression if there is significant mass effect. Radiotherapy also is not much effective. We present a boy of six year with bithalamic glioma.

## Case Report

A 6-year-old male who presented with complaints of tremors in right upper limb for two months which did not increase in frequency or severity over this period. He had history of occasional holocranial headache for two months. There was no memory or behavioral changes or compromise of school performance. Systemic examination was normal and neurological examination revealed no gross deficit except blurred margins of optic disc. Contrast enhanced CT scan showed large defined isodense mass lesion involving the bithalamic region with continuity in midline. Lateral ventricles were enlarged anteriorly and posteriorly to the mass. There was no enhancement on contrast.

MR of head showed huge bithalamic lesion, of size about 7 cm × 7 cm × 5 cm interconnected with massa intermedia without any gap in between, occupying whole of the third ventricle, except a part anteriorly. It was uniformly isointense on T1 weighted and, hyperintense on T2 and FLAIR weighted images, with no contrast enhancement.

**Figure 1:**
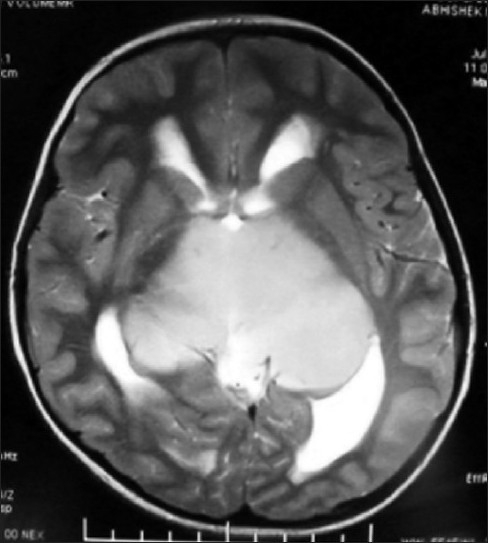
MR scan of head axial T2W1 image showing large thalamic glioma with involvement of left temporal lobe, uniformely hyperintense

**Figure 2:**
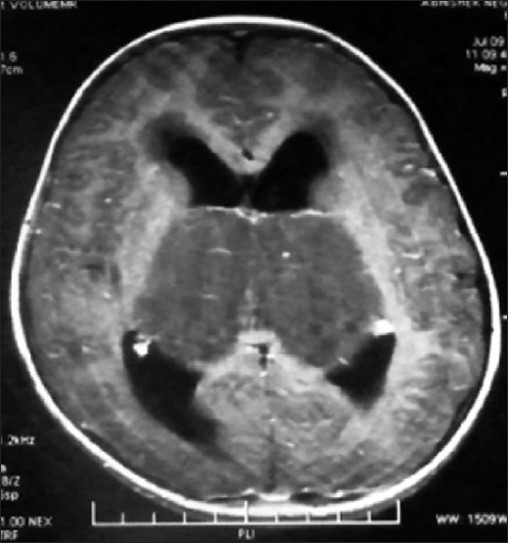
MR scan of head on gadolinium administration showing no enhancement

**Figure 3:**
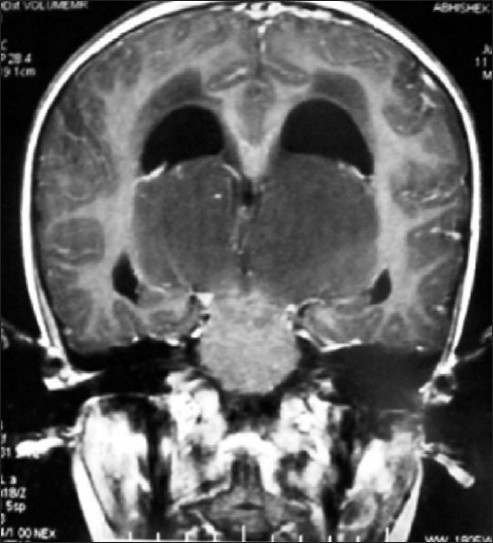
MR scan of head coronal section of enhanced MR showing limitation of tumor mainly in bilateral thalamus

**Figure 4:**
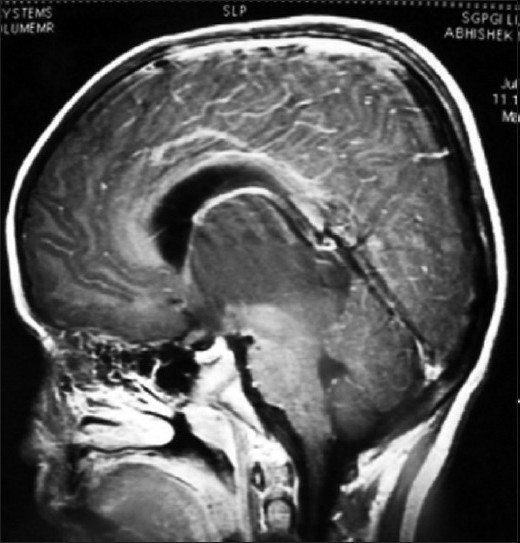
MR scan of head sagittal image showing extent of tumor till midbrain

**Figure 5:**
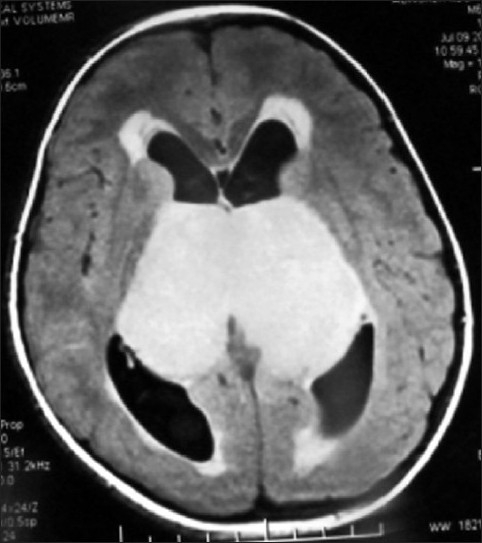
MR scan of head FLAIR image showing significant periventricular lucency with hydrocephalus

**Figure 6:**
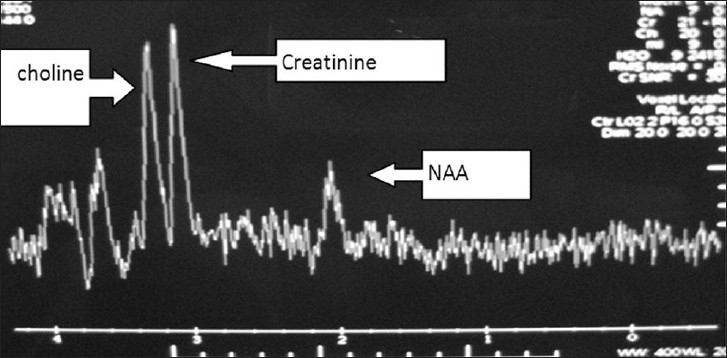
MRS of patient, showing large creatinine peak than choline

There was no restriction to diffusion weighted images. Extent of lesion was up to midbrain, infiltrating bilateral cerebral peduncles reaching to left medial and posterior temporal and medial occipital lobes. Lateral ventricles were dilated with periventricular lucency and no perilesional edema was present [Figures 1–5]. MR spectroscopy (water suppressed proton MRS) of the tumor revealed choline and creatinine peaks with creatinine peak greater than choline peak. The N acetylaspartate signal was decreased. Small lactate peak was also noticed [Figure 6].

**Figure 7:**
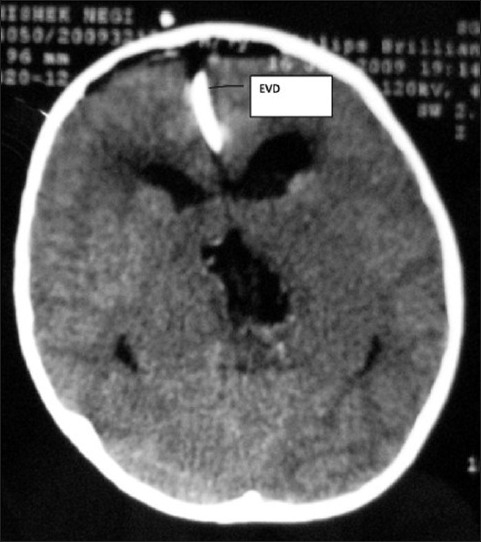
Axial section of noncontrast CT scan of head on postoperative day 1, showing external ventricular drain in situ with tumor cavity

**Figure 8:**
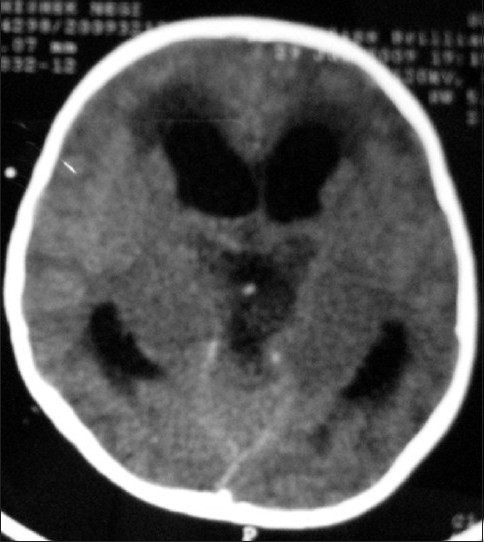
Axial section of noncontrast CT scan of head on post operative day 5 after the removal of external ventricular drain, showing enlarged size of ventricle

As the mass was very bulky and ventricles were also obstructed, a decision to debulk (and not only biopsy) was taken in order to reduce the mass effect in supratentorial compartment. He was planned for surgery with interhemispheric and transcallosal approach. Lateral ventricles were distorted. A possible tumor decompression was done and a decompression of about 30% was acheived. Intraoperatively tumor was very firm, moderately vascular which was not yielding to suction and to CUSA easily. It was so tough that the excision was probably causing trauma to bilateral thalamic nuclei on account of energetic movement of the hands and instruments. The squash was suggestive of low grade glioma. Considering the total excision impossible, an external ventricular drainage catheter was placed in right ventricle and wound was closed following a good hemostasis. Patient was put on elective ventilation in postoperative period, but it was soon weaned off. The patient deteriorated in sensorium and breathing within 6 h, elective ventilation was again required. Post-operatively the child had a stormy course in the ICU. He remained dependant on the ventilator and continued as unconscious for three weeks. He improved gradually and started opening eyes and was weaned off from the ventilator in further three weeks time. CT scan suggested an operative site cavity and an increase in size of lateral ventricles [Figures 7 and 8].

As he had high grade fever and CSF culture showed coagulase negative staphalococcus aureus growth, shunt was not done and ommaya reservoir was placed in right frontal region.

Owing to prolonged need of ventilator, tracheostomy was done. Regular aspiration of CSF was done under aseptic condition for two weeks with coverage of antibiotics according to sensitivity A biventricular shunt was done with y connector with medium pressure chhabra chamber. Patient improved after shunt. Within few days, ventilator was weaned off and decannulation was done. His histopathology showed a moderately cellular tumor composed of mild anisomorphic cells embedded in fibrillary matrix. Tumor cells displayed mild hyperchromasia and pleomorphism. Mitotic figures or endothelial proliferation or necrosis were not seen. Immunohistochemistry revealed tumor cells positive for GFAP. MIB index was low. Synaptophysin positive ganglionic cells were few. KI 67 index was < 2%. Impression was fibrillary astrocytoma (WHO Grade 2). Patient is being discharged on Ryle’s tube and planned for radiotherapy after two months. At the time of discharge, the child was conscious, following commands, breathing spontaneously, tracheostomy site healthy and had started accepting orally.

## Discussion

Thalamic gliomas are rare tumors of brain accounting about 1-1.5% of all brain tumors. Twenty-five percentage of this is reported in people of age less than 15 years. In one study incidence was reported to be 0.84 -5.2% of all intracranial tumors.[1–4] This broad discrepancy is because of the difficulty in differentiating between primary and secondary thalamic gliomas, that is, arising from adjacent structures like caudate nuclei, brain stem or pineal gland. Primary bithalamic gliomas are rarer. About fifty cases have been reported in literature till now. In pediatric age group only 15 cases have been reported.[2,5]

Bilateral thalamic tumors are different from their counterparts presenting as unilateral thalamic gliomas. These have poor prognosis in spite of benign histopathology.[1,2] There are two views regarding the origin of bilateral thalamic glioma.[6–8] According to one view, these are supposed to arise on one side of thalamic nuclei and spread to other side with time, while according to other view they arise by sprouting of tumors from subependymal region of the third ventricle. In an autopsy series,[8] a connection was found across midline through the posterior part of corpus callosum and prerubral region of midbrain; however, this hypothesis is difficult to accept because of topographic characteristics of these lesions. These are bilaterally symmetrical, confined within the thalamic nuclei, and not violate the border between gray and white matter. Commonly these tumors do not involve midbrain, pineal gland and midline basal subependymal region of third ventricle and only in late phase involve temporal lobe through connection with amygdaloid nuclei[7] or brain stem[6] as in our case where there was involvement of left side of temporal lobe.

Age of presentation varies from 3 months to 70 years,[2,5] but is less common in pediatric age group.[2] Few cases have been reported below six years of age.[2]

Presentation is also quite typical, symptoms and signs are mild even though the patients may have large tumors.[3] Raised intracranial pressure is generally not because of hydrocephalus, as hydrocephalus is usually absent or mild.[2] (unlike in our case where hydrocephalus was significant); however, lesion in itself causes mass effect. Various clinical presentations can be explained with the involvement of different nuclei or tracts of this region. Hemiparesis, sensory disturbances, dysmetria, unsteady gait, torticolis, and nystagmus are the usual presenting features. In adults, personality changes are more common in comparison to focal neurological deficit. Anatomical substrates involved in dementia and personality changes are dorsomedial nuclei of thalamus and their connections with frontal and temporal lobes; for memory dysfunction and disorientation, the nuclei are anterior thalamic nuclei and mammillothalamic tracts. Frontal lobe dysfunction results from infiltration of midline and intralaminar nuclei, which connect with prefrontal cortex, a part of the reticular activating ascending tracts of the cerebral cortex. Loss of psychic self activation is because of the involvement of striatal ventral pallidal thalamic fronto mesial limbic loop at different levels. Sensory dysfunction is attributed to the involvement of ventral nuclei.[9] Our case is peculiar, as he presented with raised intra-cranial pressure and tremors only, which is quite uncommon particularly for the large size of the bithalamic tumor which was encountered in our case. Tremors can be explained with infiltration of ventroanterior and ventrolateral thalamic nuclei and dentate rubrothalamic tract.[2]

On CT scan, these tumors appear as bilateral symmetrical isodense nonenhancing lesions, with little or no mass effect. MRI is the best radiological investigation in which the lesion appears as hypo to iso intense on T1 weighted images and hyperintense on T2 weighted images with no enhancement on contrast, respecting the grey white boundaries and confined to thalamus. Hydrocephalus is usually mild, but in our case was significant hydrocephalus with periventricular lucency. It was obviously because of the obstruction of the third ventricle and compression of the bodies of the lateral ventricle. Differential diagnosis to be kept in thalamic region are vascular lesions(deep cerebral venous thrombosis), other tumors (germinomas, teratomas, and lymphomas), infections (viral encephalitis, toxoplasmosis and abscesses and metabolic disorders).[10] MR spectroscopy is helpful in differentiating bithalamic gliomas from other lesions.[10] MR spectroscopy is peculiar, and suggesting that primary bithalamic glioma are different from unilateral thalamic glioma,[6] in respect to metabolic pattern, though histopathology may be similar. MRS showed enlarged –creatinine and choline peak, (creatinine peak greater than choline peak) with decreased peak of NAA, which is suggestive of higher cellular metabolism(creatinine peak) as well as higher proliferative potential(choline peak), unlike to low grade gliomas where choline peak is greater than creatinine peak while neuronal loss is same as in low grade glioma, suggested by the decrease in NAA peak in both.

Owing to diffuse and bilateral involvement of thalamus, surgical excision is difficult and no case of radical removal has been reported,[1–3,5,10] so role of surgery is only to get tissue for histological diagnosis, which can be obtained by open surgery with different approaches such as interhemispheric transcallosal, infratentorial supracerebellar, or transsylvian transinsular approach[11,12] or endoscopically or under stereotactic guidance.[1,11,12] But in cases where there is a large tumor causing much mass effect, debulking should be tried. Although we tried to debulk the tumor, it was not achieved to the desired level. In case of hydrocephalus, biventricular shunt is an option. Most cases were low grade gliomas (grade 2 of WHO classification). Role of adjuvant radiotherapy and chemotherapy is not established. Outcome is poor irrespective of treatment.

## Conclusion

Bilateral thalamic glioma are rare neoplasms. They are different from unilateral neoplasm, with respect to neuroradiological and metabolic aspect and prognosis. Even though they are benign, prognosis is poor, which may be because of bilateral involvement of thalamic nuclei. Even large bithalamic gliomas may present with minimal symptoms. An obstructive hydrocephalus because of compression of the third ventricle and bodies of the lateral ventricle may cause dilatation of ventricles proximal and distal to the mass. Post-operative course after debulking may be stormy.
